# Accelerated MR thermometry using the Kalman filter

**DOI:** 10.1186/2050-5736-3-S1-P40

**Published:** 2015-06-30

**Authors:** Li Zhao, Samuel Fielden, Wilson Miller, Xue Feng, Max Wintermark, Kim Butts Pauly, Craig Meyer

**Affiliations:** 1 University of Virginia, Charlottesville, Virginia, United States; 2 Stanford University, Stanford, California, United States

## Background/introduction

Magnetic resonance (MR) imaging plays an important role in monitoring thermal treatment. It can quantify thermal dose with temperature maps based on the proton-resonance frequency shift. Volumetric coverage is desirable, but acquiring multiple slices imaging is time consuming. Therefore, accelerated methods are needed to improve the spatial and temporal resolution in MR thermometry. Multi-channel coils are not widely available for MR-guided FUS systems, so conventional parallel imaging methods cannot be used for acceleration. Compressed sensing methods show promise, but the computation is currently too slow to provide real-time feedback. The Kalman filter is an optimal estimation method that has been widely used for real-time tracking in other fields. It has been studied for filtering of temperature for FUS. Here we apply it to accelerate image acquisition for thermometry.

## Methods

The Kalman filter (KF) uses prior state information to predict the current state with a dynamic system model:

x(k) = x(k-1) + w(k-1)

z(k) = U(k) F x(k) + v(k)

x(k) is the target image at the kth frame and the first function describes the state transition. z(k) is the corresponding acquired data. F is a Fourier transform operator and U(k) is an undersampling pattern. w and v are the system and measurement noise, assumed to have white Gaussian distributions with covariance matrices estimated by the KF. w models state changes resulting from heating. A numerical phantom was used to validate the proposed method. One normalized slice was sampled with a 128x128 matrix. The focal spot followed a 2D Gaussian distribution spatially. The temperature evolves with exponential increase and decay, with 15-degree peak. 100 image frames were simulated with complex Gaussian noise (std = 0.01). A gel phantom was tested with a HIFU system (RK-100, FUS Instruments Inc., Toronto) in a 3T Siemens Trio. Fully sampled data were acquired by a gradient echo sequence with 64x64 matrix, FOV 64mm×64mm and resolution 1mm×1mmx5mm. TR/TE = 15/6 ms and bandwidth 500 Hz provided temporal resolution 0.96s per frame. The sequence acquired data continuously during three consecutive 30-second intervals corresponding to baseline, continuous sonication, and cooling. Data were undersampled by a factor of 2 along the phase encoding direction (y) and reconstructed by zero filling, view sharing, KF, and KF with first frame initialized by view sharing. Temperature maps were calculated by the PSF method. The temperature map of fully sampled k-space was chosen as the standard to evaluate the performance of the above methods.

## Results and conclusions

**Figure 1:**
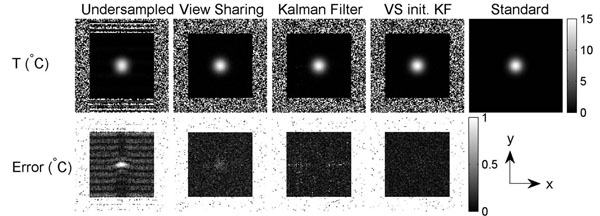
Simulated accelerated spatial temperature maps. The fully sampled noiseless temperature map is shown as standard. Absolute error maps show the KF method reduced undersampling artifacts and estimation error in the temperature map.

**Figure 2:**
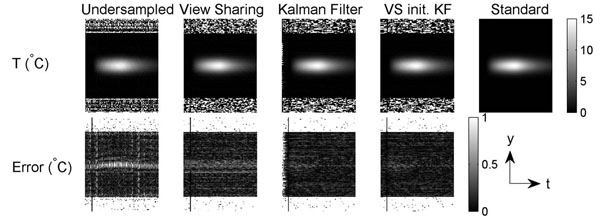
Simulated accelerated temporal temperature maps. The fully sampled noiseless temperature map is shown as standard. The KF method reduced aliasing and error. Initialized by view sharing, KF further reduced the error in the first few frames.

**Figure 3:**
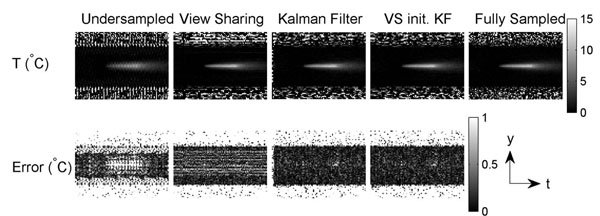
Experimental accelerated temporal temperature maps. The fully sampled temperature map is shown as reference. The KF method reduced aliasing and error. Initialized by view sharing, KF further reduced the error in the first few frames.

**Figure 4:**
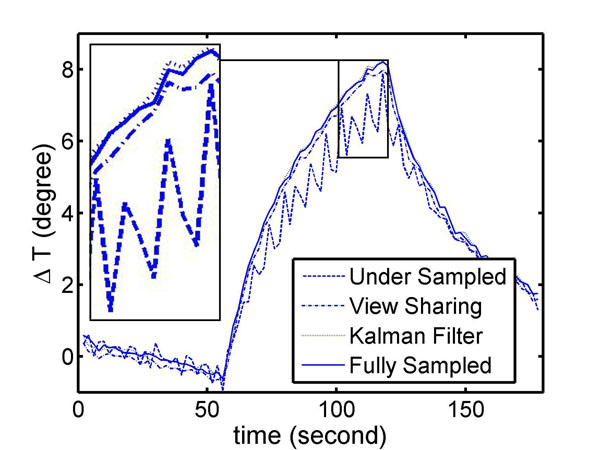
Temporal plot of focal spot in gel phantom. ROIs (3x3 pixels) were selected around the highest temperature. The average temperature shows the KF method approximated the temperature more accurately than other methods with 2X acceleration.

Figs. 1 and 2 show the simulated spatial and temporal temperature maps. The KF method produced negligible aliasing artifacts in the temperature map (top) and resulted in better approximation of the standard temperature with less error (bottom). With the first image initialized by view sharing, KF further reduced the error in the first few frames. Fig. 3 shows the experimental temporal temperature maps. Fig. 4 shows the temporal profile of the focal spot (3x3 pixels). The KF method approximated the fully sampled image accurately and provided a temperature map with less error. In conclusion, the KF method can estimate temperature accurately with a speed-up of at least 2X, enabling real-time thermometry with greater spatial coverage.

